# Study of microstructure and fracture properties of blunt notched and sharp cracked high density polyethylene specimens

**DOI:** 10.1038/s41598-017-03884-6

**Published:** 2017-07-21

**Authors:** Huanyu Pan, Sheila Devasahayam, Sri Bandyopadhyay

**Affiliations:** 1 School of Materials Science & Engineering, University of New South Wales, Sydney, Australia; 2 Department of Environmental Sciences, Macquarie University, Sydney, Australia

## Abstract

This paper examines the effect of a broad range of crosshead speed (0.05 to 100 mm/min) and a small range of temperature (25 °C and 45 °C) on the failure behaviour of high density polyethylene (HDPE) specimens containing a) standard size blunt notch and b) standard size blunt notch plus small sharp crack – all tested in air. It was observed that the yield stress properties showed linear increase with the natural logarithm of strain rate. The stress intensity factors under blunt notch and sharp crack conditions also increased linearly with natural logarithm of the crosshead speed. The results indicate that in the practical temperature range of 25 °C and 45 °C under normal atmosphere and increasing strain rates, HDPE specimens with both blunt notches and sharp cracks possess superior fracture properties. SEM microstructure studies of fracture surfaces showed craze initiation mechanisms at lower strain rate, whilst at higher strain rates there is evidence of dimple patterns absorbing the strain energy and creating plastic deformation. The stress intensity factor and the yield strength were higher at 25 °C compared to those at 45 °C

## Introduction

High density polyethylene (HDPE) finds applications in high modulus/ high load bearing and long exposure to harsh condition applications typically in ambient temperature ranging between 25 to 45 °C. HDPE is a semi-crystalline polymer, where the modulus depends on the degree of crystallinity.

Normally, the mechanical properties of polymers exhibit high dependence on temperature, strain rate and modulus due to their viscoelasticity nature (Kinloch and Young^1^; Coates and Ward^2^ George, Thomas *et al*.^3^ Desari and Misra^4^, Mae^5^, Plaseied and Fatemi^6, 7^). Change in Young’s modulus with temperatures in polymers is marked by five distinct transitions regions, with the prominent transition at the glass transition temperature (Sperling)^8^. According to time-temperature physical equivalence (Van Krevelen and Nijenhuis)^9^, lower temperatures correspond to high strain rate and vice versa. This implies that increasing strain rate or decreasing temperature will have similar effects on mechanical/fracture behaviour of thermoset polymers (Pan and Bandyopadhyay)^10^. As the glass transition temperature of HDPE is well below the room temperature, it is categorised as a ductile material. Consequently the fracture properties of HDPE would normally be governed by fracture energy and not the fracture toughness.

Two basic types of fracture in materials are a) brittle fracture and b) ductile fracture. A material absorbs more energy when it fractures in a ductile fashion than in a brittle fashion. Fracture in polymeric materials may be ductile or brittle depending on strain rate, the stress system and the temperature. Both types of fracture may be observed in one material depending on the service conditions. When the temperature is significantly reduced, polymeric materials may show transition from ductile to brittle behaviour.

The fracture properties can be determined using specimens with either blunt notch or sharp crack at the notch tip. When a sharp notch is used the energy necessary to initiate a crack is small, whilst for a blunt notch the crack initiation energies are higher. A sharp crack sample is reportedly affected by the temperature compared to a blunt notched sample (Chanda and Roy)^11^. Transition to a brittle fracture in sharp crack sample is attributed to the local triaxial stress concentration increasing the local rate of strain at the notch tip. In a blunt notch sample both crazing and shear yielding mechanisms may operate simultaneously.

This paper investigates the mechanical and micro-deformation failure aspects as affected by strain rate and temperatures in HDPE tensile specimens containing a) single edge blunt notch, and b) single edge sharp crack.

## Experimental

### Samples

**Figure 1:**
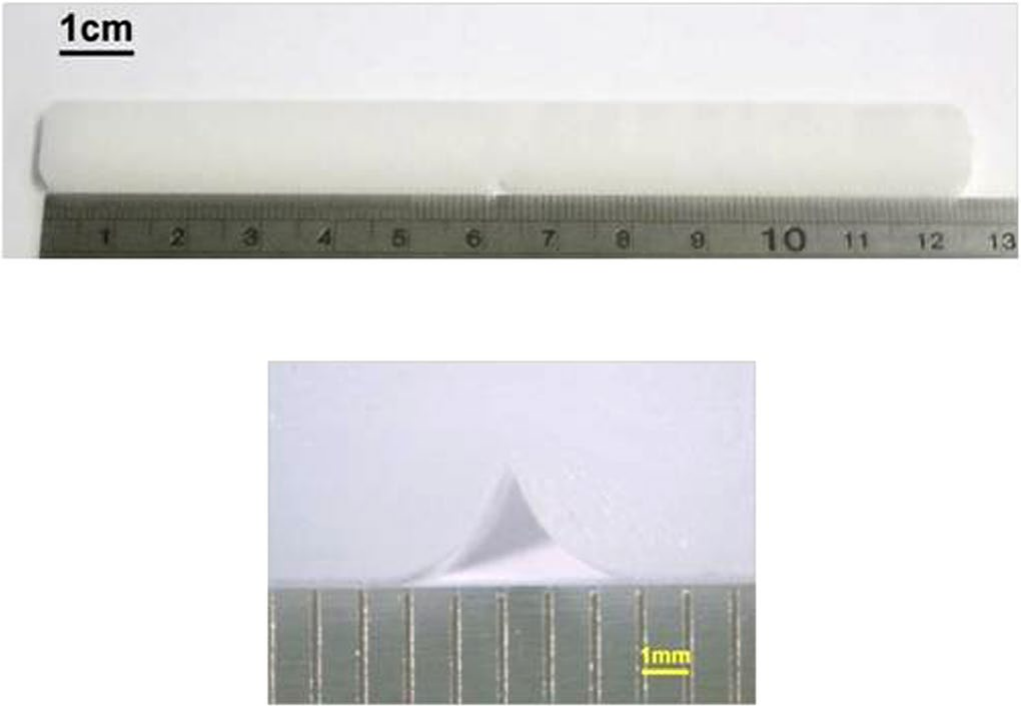
Image of HDPE tensile test specimen with moulded-in blunt notch from the tip of which sharp crack can also be made by a razor blade.

High density polyethylene GD 7255, HOECHST were used in the present study. Test specimens (Fig. 1) are injection moulded (model Boy 15 S) into notched strips, approximately 127 mm long, 12.5 mm wide, 4 mm thick with a single-edge notch of depth 2.5 mm (notch-tip radius 0.25 mm – similar to Charpy specimens) in the middle of the length. These specimens were used as ‘blunt-notched specimens’ whilst another set of specimens were used with a sharp crack induced by a new razor blade at the tip of the notch, (mode I loading) as is common in plastics fracture testing (Bandyopadhyay & Brown^12, 13^, Gong & Bandyopadhyay^14^.

### Tensile Test

Tensile tests were performed in an Instron 5565 universal screw machine (5 KN capacity) at crosshead speeds of 0.05, 0.5, 5, 50 and 100 mm/min. The experiments were carried out at 25 °C and 45 °C, well below the melting point of the HDPE and much above the glass transition of HDPE.

### Optical Microscopy

The tensile tested HDPE fractured specimens, cut approximately 5 mm below fracture surface by hand saw, were studied using a low magnification optical microscopy (OM) and stereo zoom microscopy (model Olympus SZ-STU2,) to observe micro-appearance of the fracture surfaces.

### Scanning Electron Microscopy

A scanning electron microscope (SEM) (model Hitachi TM3000) was used to observe micro-appearance of the fracture surfaces of the failed HDPE samples. Following optical microscopy examination, specimens were gold coated (by a sputter coater, model Leica EM SCD050) at high vacuum under a current of 60 mA for 20 seconds. The thickness of gold coating was approximately 30 nm.

## Results and Discussion

### CHS effects at room temperature, 25 °C

**Figure 2:**
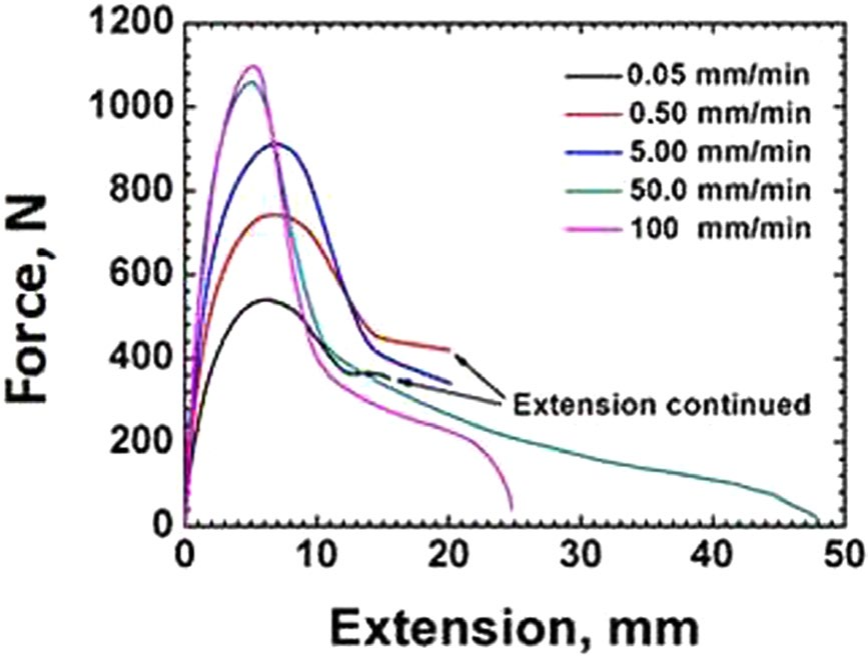
Load-extension curves of blunt notched HDPE at 25 °C under various crosshead speeds.

**Figure 3:**
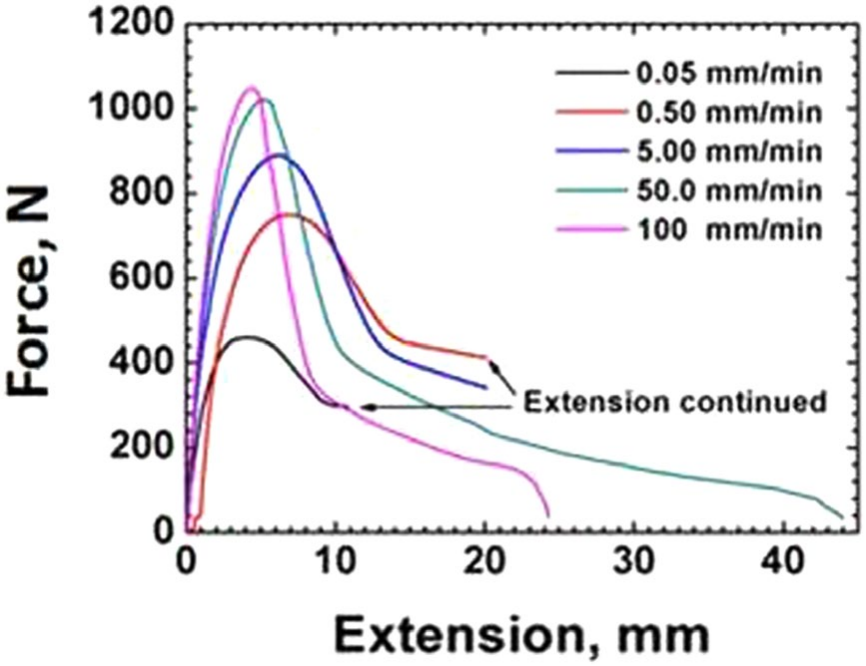
Load-extension curves of sharp crack HDPE at 25 C under various crosshead speeds.

The tensile test results recorded as load-extension curves under various crosshead speeds c are shown in Fig. 2 for blunt notched HDPE samples. As can be seen from the Fig. 2, the maximum load was recorded for the highest cross head speed (CHS). Figure 3 shows the load-extension curves at various crosshead speeds at 25 °C for sharp notched HDPE, where a similar behaviour is observed, that is maximum load at the highest CHS.

A semicrystalline polymer such as HDPE exhibits more of a ductile behaviour. At high crosshead speed corresponding the strain to failure is much lower in both types of samples indicating close to- brittle type failure at high strain rates. A ductile behaviour at lower strain rates is evident from the longer extension for both blunt notch and sharp crack samples as was also observed in polymers by Chanda and Roy^15^, and Perkins^16^. This is attributed to the viscoelastic nature of the polymer, which under high strain rate, or low temperature, tends to exhibit brittle or close-to-brittle failure (Pan and S. Bandyopadhyay^9^, O’Connell *et al*.^17^).

**Figure 4:**
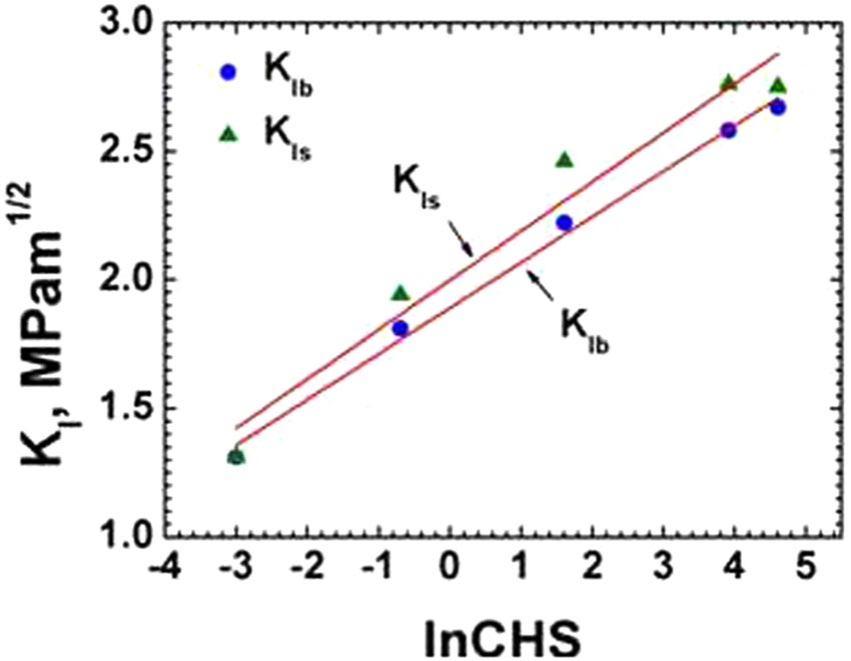
Plot of anticipated KIb and KIs as a function of ln CHS at 25 °C.

A hypothetical failure stress intensity factor K, for blunt notch (K_Ib_) and sharp notch (K_Is_) calculated according to equation 1 (Broek)^18^, was plotted against ln CHS. The results are are shown in Fig. 4 and Table 1 indicating a linear dependence of blunt notch K_Ib_ and sharp crack K_Is_ on the ln CHS varying between 0.05 mm/min to 100 mm/min.

1 $${{\rm{K}}}_{{\rm{I}}}={{\rm{Pa}}}^{1/2}[1.99-0.41({\rm{a}}/{\rm{W}})+18.7{({\rm{a}}/{\rm{W}})}^{2}-38.48{({\rm{a}}/{\rm{W}})}^{3}+53.85{({\rm{a}}/{\rm{W}})}^{4}]/{\rm{BW}}$$

where, P = maximum load, a = considered as initial notch (or crack) length, W = thickness, B = width.

**Table 1:** ΔK_I_ and Δσ_y_ values for blunt and sharp notched HDPE at various crosshead speeds.

CHS, mm/min	0.05	0.50	5.00	50.0	100
ΔK_I_ = K_Ib_ − K_Is_, MPam^1/2^	0.00	−0.13	−0.24	−0.18	−0.08
Δσ_y_ = σ_yb_ − σ_ys_, MPa	1.61	0.15	0.43	0.74	1.00

**Figure 5:**
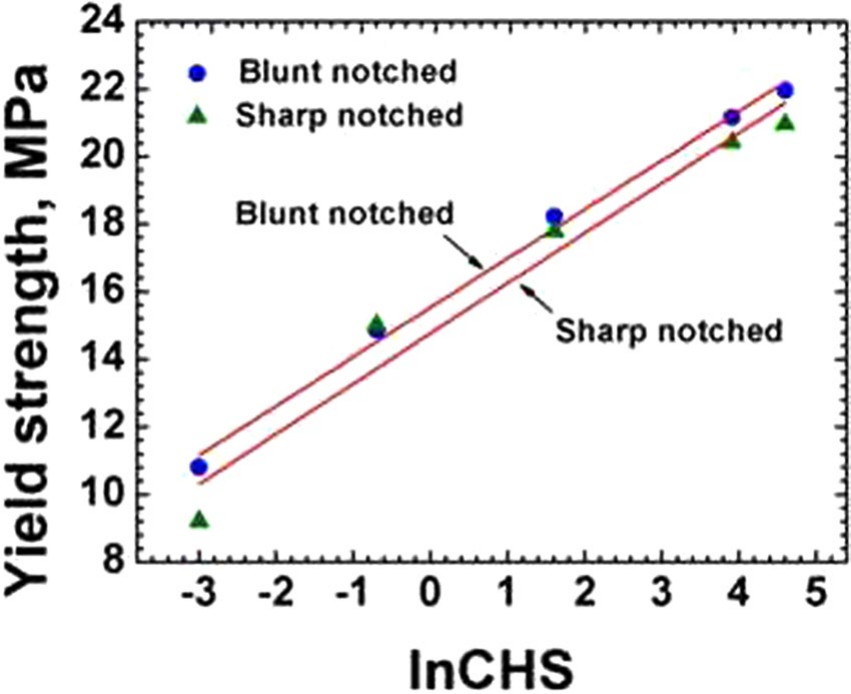
Yield strength of blunt notch and sharp crack specimens as a function of ln CHS at 25 °C.

Figure 5 shows the logarithmic dependence of yield strength σ_y_ on the CHS for both sharp crack and blunt notches (Table 1). The yield strength for blunt notch increased from 10.8 to 21.95 MPa (103% increase) and for sharp crack σ_y_ increased from 9.19 to 20.95 MPa (128% increase) as crosshead speed increased from 0.05 to 100 mm/min. As seen in Figs 4 and 5 both K_Ib_ and σ_y_ of blunt notch and sharp crack HDPE have a logarithmic relation to CHS. The K_Ib_ or K_Is_ and σ_y_ of HDPE did not show significant difference between the sharp crack and blunt notched samples – which indicates that the blunt notch did not generate any effective stress concentration to cause any brittle failure because of the high ductility of the material at 25 °C. Also the 0.25 mm sharp crack tip was quite ductile for the HDPE material – whereas for more brittle polymers such as epoxy resins, normal and toughened, it may have significant effect [Bandyopadhyay S^19^].

The sharp crack and blunt notch show no significant distinction in their K and σ_y_ values, even though it is expected that the blunt notches show lower yield strength and fracture property compared to the sharp crack owing to the increased plastic flow at notch tip.

### Moderate High Temperature Effects at 45 C

**Figure 6:**
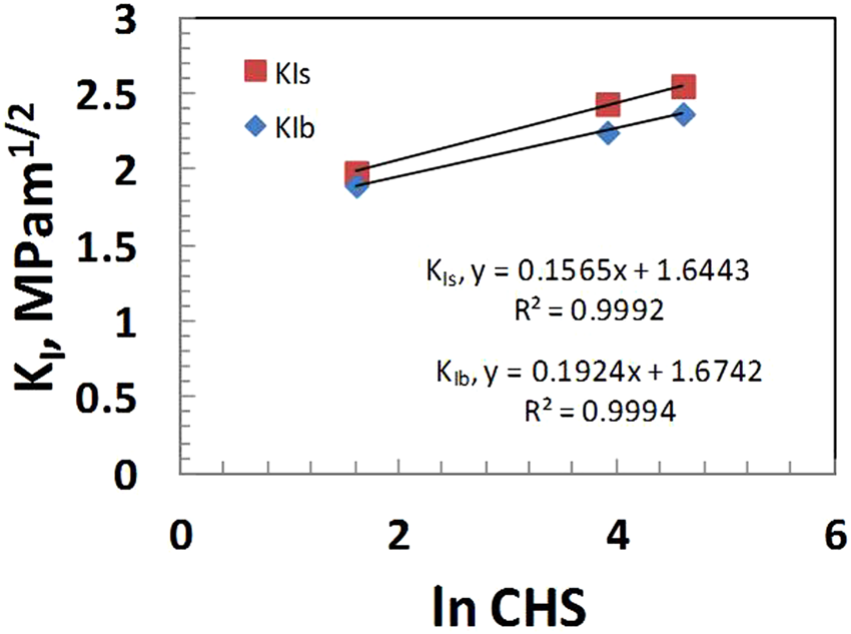
Plot of KIs as a function of ln CHS for sharp and blunt crack samples 45 °C.

**Figure 7:**
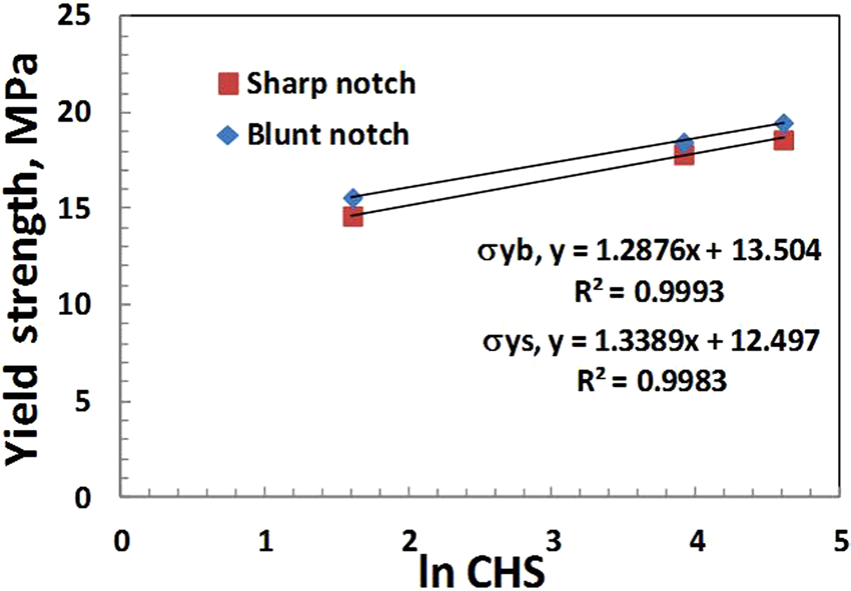
Plot of Yield strength of sharp and blunt crack HDPE as a function of ln CHS at 45 °C.

Figures 6 and 7 show the values of KI and σy on the CHS at 45 °C for both the sharp crack and blunt notch specimens. Figures 4, 5, 6 and 7, indicate increasing linear trends of KI and σy with increasing ln CHS at 25 °C and 45 °C.

K_Ib_ and K_Is_ at 45 °C are lower than the K_Ib_ and K_Is_ at 25 °C which is understandable, because a 20 °C higher temperature would soften the polymer chains, by some amount. The K_Is_ at 45 °C and 25 °C showed similar stress intensity variation with respect to CHS values for sharp crack samples with the slope values equal to 0.19. However, for blunt samples the stress intensities are almost the same at 45 °C, (0.18 to 0.16 MPam^0.5^. The σ_y_ at 45 °C for both blunt and sharp notches are lower than that at 25 °C, as expected in a semicrystalline thermoplastic [Sperling]^8^. This is because σ_y_ at 45 °C is lower than at 25 °C as at higher temperature the yield stress is lower and consequently K_Ib_ and K_Is_ at 45 °C are also lower compared to those at 25 °C.

### Crack length-Optical Microscopy study

**Figure 8:**
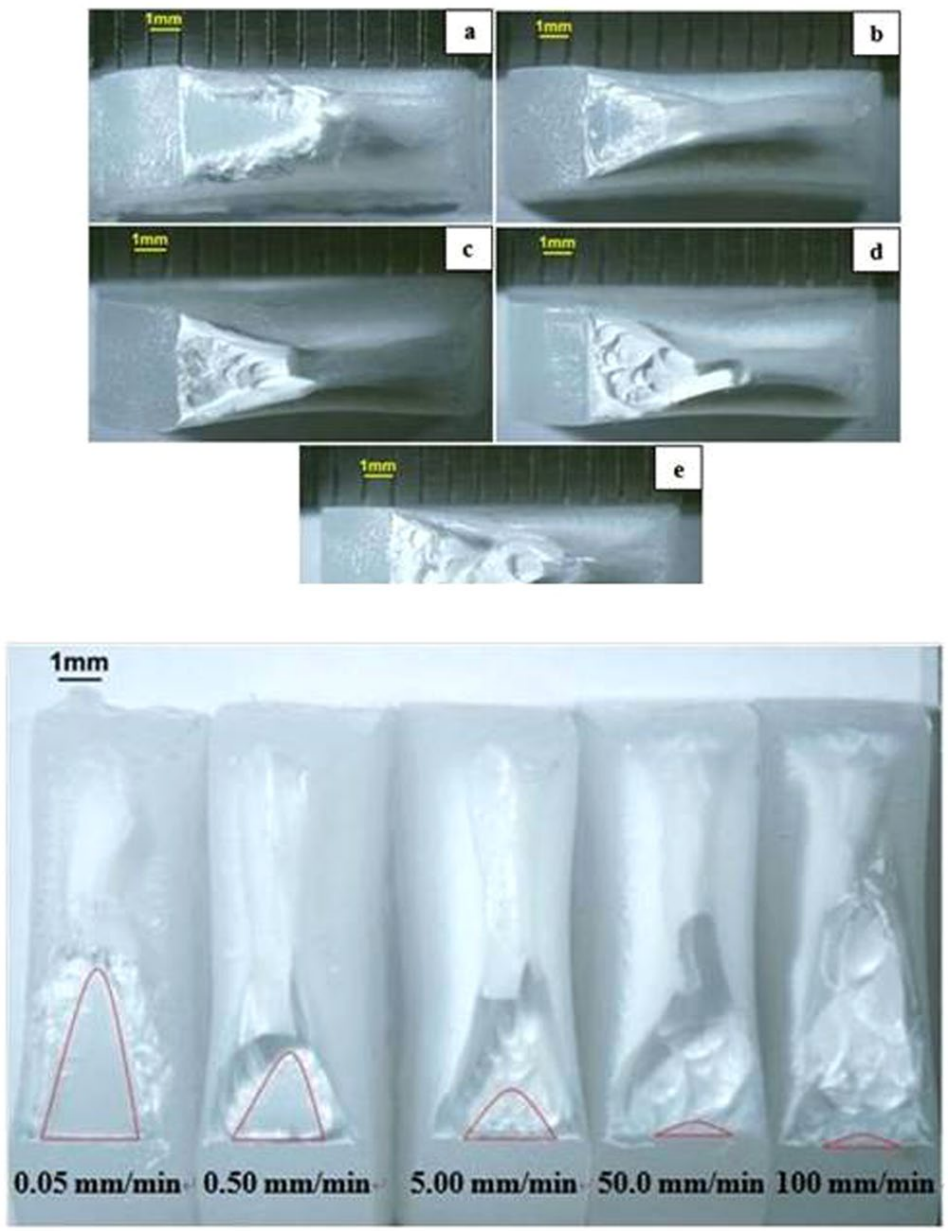
(**a**) Optical microscopy images of blunt notched specimens at various crosshead speeds (notch at the left, crack propagating from left to right). (**a**) 0.05 mm/min; (**b**) 0.50 mm/min; (**c**) 5.00 mm/min; (**d**) 50.0 mm/min; (**e**) 100 mm/min at 25 °C. (**b**) Critical crack length of blunt notched specimens seen in optical microscopy (notch at the bottom, crack propagating from bottom to top) (**a)** 0.05 mm/min; (**b**) 0.50 mm/min; (**c**) 5.00 mm/min; (**d**) 50.0 mm/min; (**e**) 100 mm/min at 25 °C.

The stereo zoom microscopy images of the fracture surfaces of blunt notched HDPE after tensile testing are shown in Fig. 8a and b. As displayed in Fig. 8a, notch tip is at the left and crack propagates from left to right. Images (a), (b), (c), (d) and (e) respectively represent fracture surfaces induced at crosshead speeds of 0.05, 0.5, 5, 50 and 100 mm/min. Figure 8b indicates the critical crack length of blunt notched HDPE, crack propagating from bottom to top. The critical crack length values are given in Table 2.

**Table 2:** Critical crack length of blunt notched HDPE at various crosshead speeds.

CHS, mm/min	0.05	0.50	5.00	50.0	100
**ln CHS**, **mm/min**	−2.9957	−0.6932	1.6094	3.9120	4.6052
**Critical crack length**, **mm**	4.033	1.796	1.016	0.323	0.273

**Figure 9:**
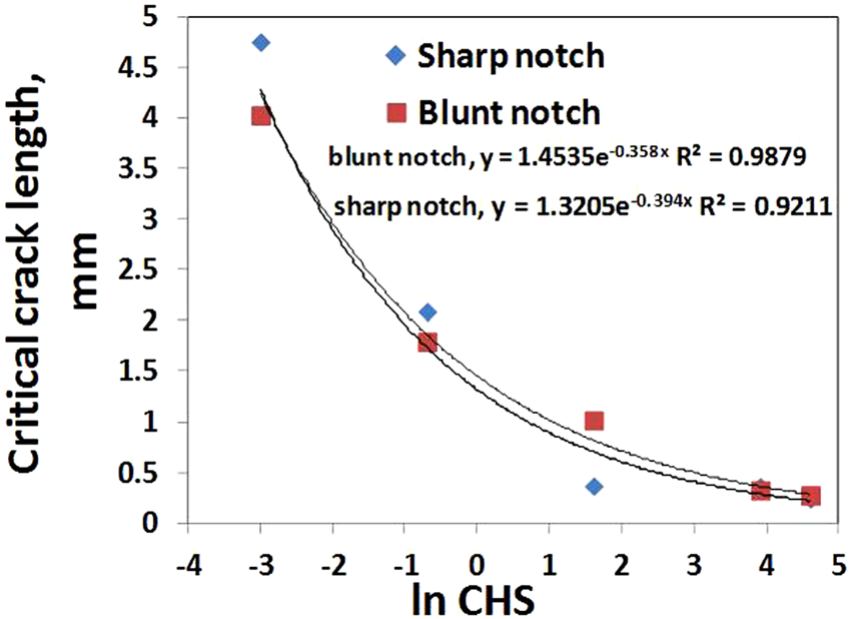
Plot of critical crack length of blunt notch and sharp crack specimens as a function of ln CHS at 25 °C

As seen in Fig. 8b, fracture surfaces of (a) and (b) exhibit similar feature, that is, both of them have a smooth parabola slow crack growth zone near the notch tip and the size of the parabola zone diminishes as crosshead speed increases from 0.05 to 0.5 mm/min. On the other hand, (c), (d) and (e) in Fig. 8b show similar uneven fast growth region following a hardly seen slow growth region near the notch tip. Slow crack growth region [Gong and Bandyopadhyay]^14^, can be determined by measuring the size on these stereo microscopy images (Fig. 8b). Figure 9 shows the exponential decrease in critical crack length as crosshead speed increases according to: y = 1.4535exp (−0.358x), R^2^ = 0.9879, for blunt notched samples and y = 1.3205 exp (−0.394x), R^2^ = 0.99 for sharp notched samples.

**Figure 10:**
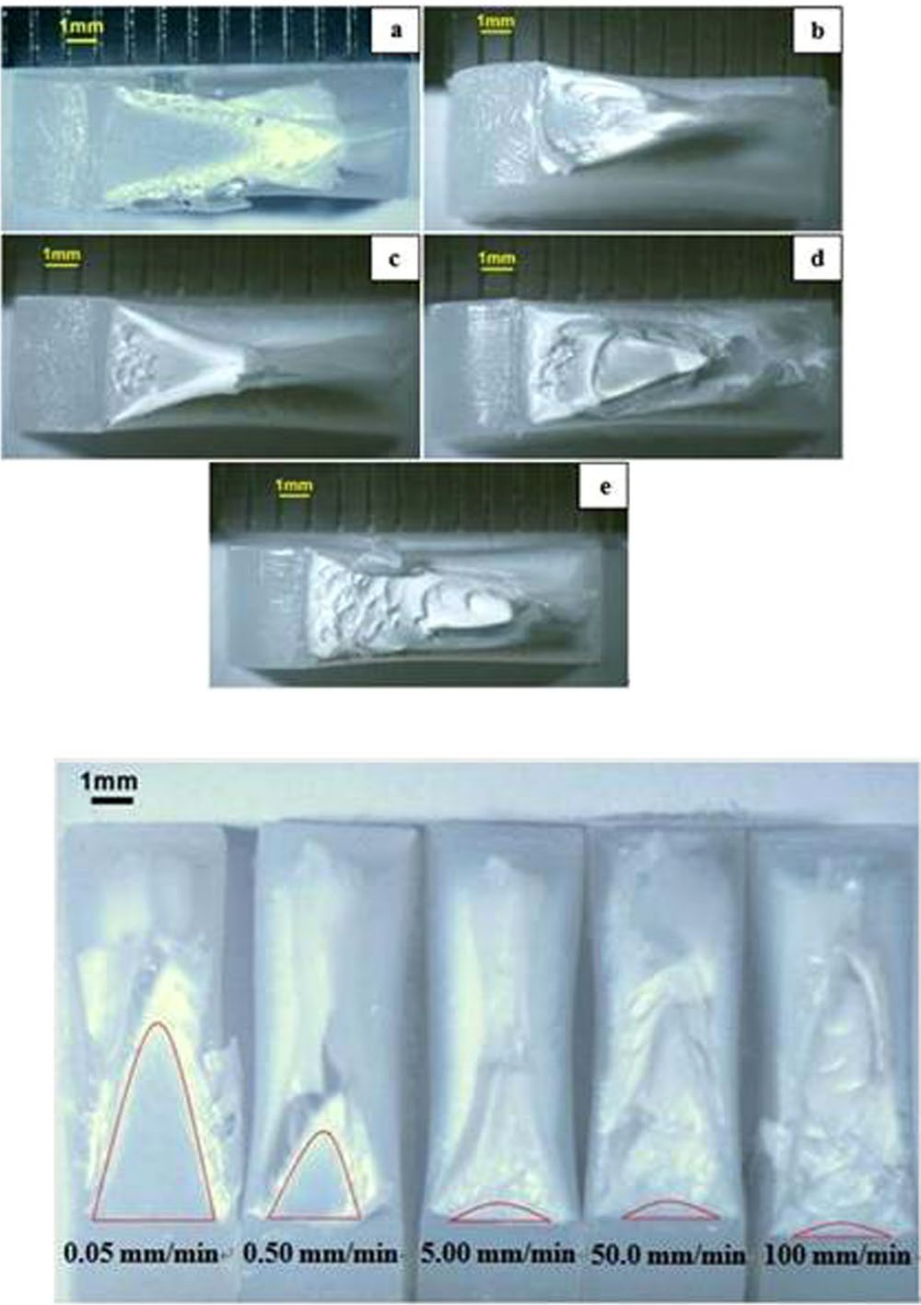
(**a**) Optical microscopy images of sharp crack specimens at various crosshead speeds (notch at the left, crack propagating from left to right). (**a**) 0.05 mm/min; (**b**) 0.50 mm/min; (**c**) 5.00 mm/min; (**d**) 50.0 mm/min; (**e**) 100 mm/min at 25 °C. (**b**) Critical crack length of sharp crack specimens seen in optical microscopy (notch at the bottom, crack propagating from bottom to top) at 25 °C.

Figure 10a shows the fracture surfaces of sharp crack HDPE where notch tip is at the left and crack propagates from left to right. Images (a), (b), (c), (d) and (e) in Fig. 10a respectively represent fracture surfaces induced at crosshead speeds of 0.05, 0.5, 5, 50 and 100 mm/min. One group consists of images (a) and (b), where there is a smooth parabola of slow crack growth near the notch tip and the size of the parabola zone reduces as crosshead speed increases from 0.05 to 0.5 mm/min. The other group is (c), (d) and (e), showing similar uneven fast growth regions with a very small (almost non-existent) slow growth region near the notch tip.

Figure 10b indicates the critical crack length of sharp crack HDPE, crack propagating from bottom to top. The exact values are given in Table 3.

**Table 3:** Critical crack length of sharp notched HDPE at various crosshead speeds.

CHS, mm/min	0.05	0.50	5.00	50.0	100
**ln CHS**, **mm/min**	−2.9957	−0.6932	1.6094	3.9120	4.6052
**Critical crack length**, **mm**	4.761	2.091	0.367	0.360	0.242

In summary, fracture surfaces of both blunt and sharp cracked HDPE have parabola type slow crack growth zone near notch tip at 0.05 and 0.5 mm/min, whilst at 5, 50 and 100 mm/min, they have similar uneven fast growth region following slow growth region. In addition, critical crack lengths of blunt notch and sharp cracked HDPE exponentially decrease as crosshead speed increases. In Fig. 9, the exponential fitting curve of blunt notched HDPE is very close to that of sharp crack HDPE and these two fitting curves cross at crosshead speed of 0.5 mm/min. Below 0.5 mm/min, sharp crack HDPE has higher critical crack length, however, blunt notched HDPE samples have a little bit higher value when crosshead speed is above 0.5 mm/min. It is inferred from Fig. 9 that the decrease in critical crack length with increasing CHS occurs at a slightly higher rate for the blunt notched samples compared to the sharp notched samples.

### Scanning Electron Microscopy Results and Discussion

#### Blunt Notched Specimens

**Figure 11:**
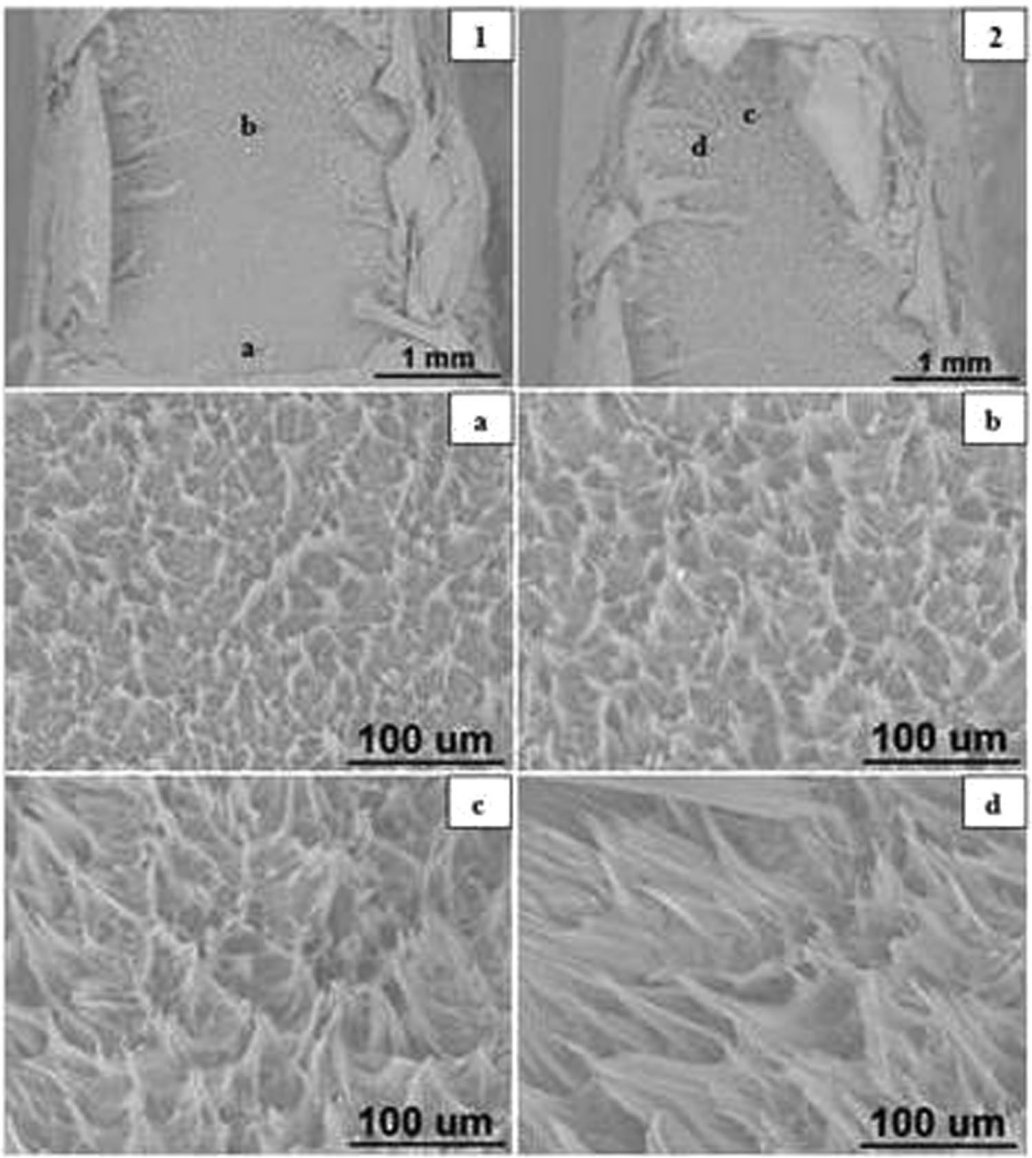
SEM images of blunt notched HDPE fracture surfaces at 0.05 mm/min (1) notch at the bottom, crack propagating from bottom to top; (2) top of propagation at 25 °C.

**Figure 12:**
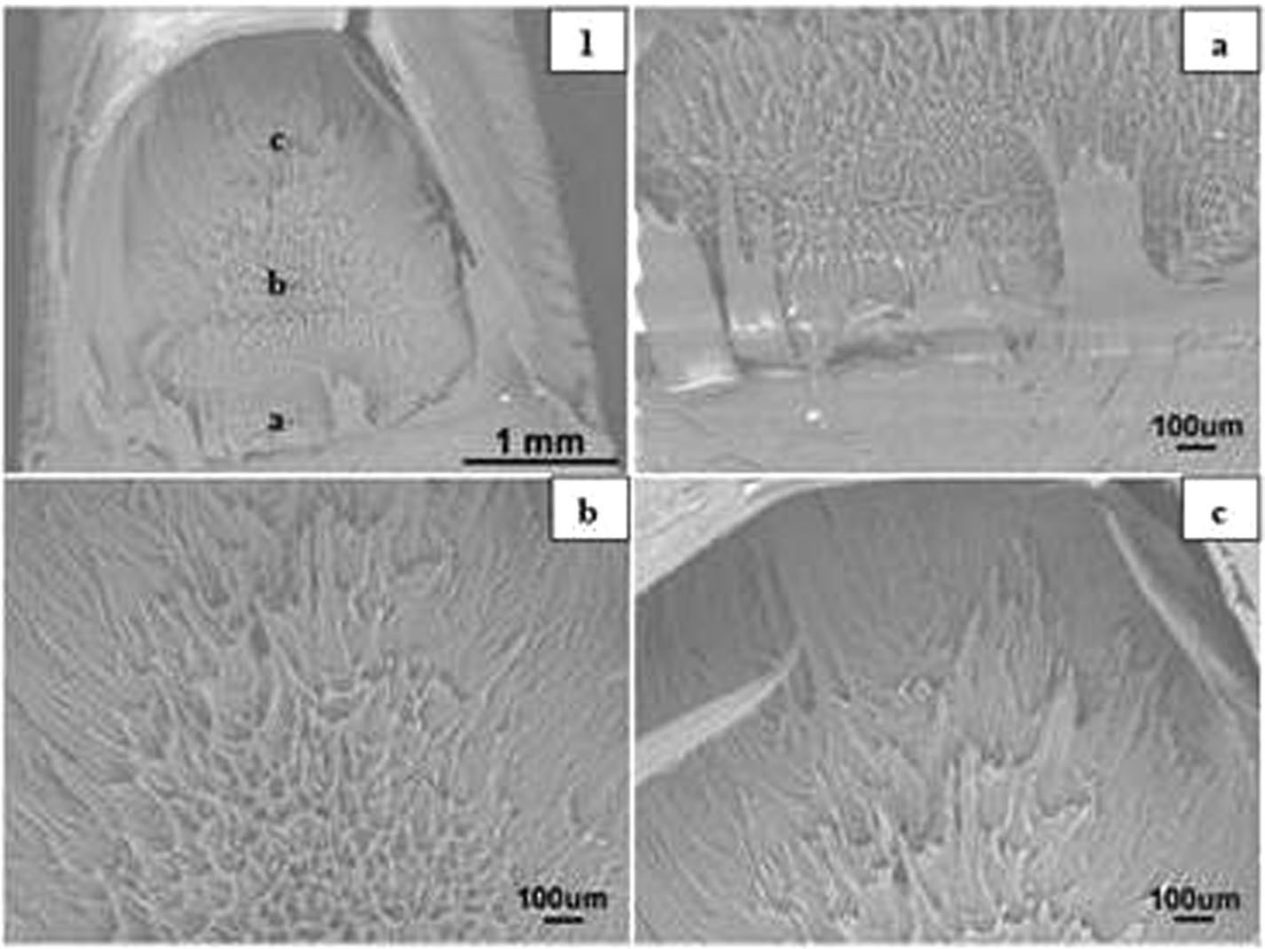
SEM images of blunt notched HDPE fracture surfaces at 0.50 mm/min (1) notch at the bottom, crack propagating from bottom to top at 25 °C.

**Figure 13:**
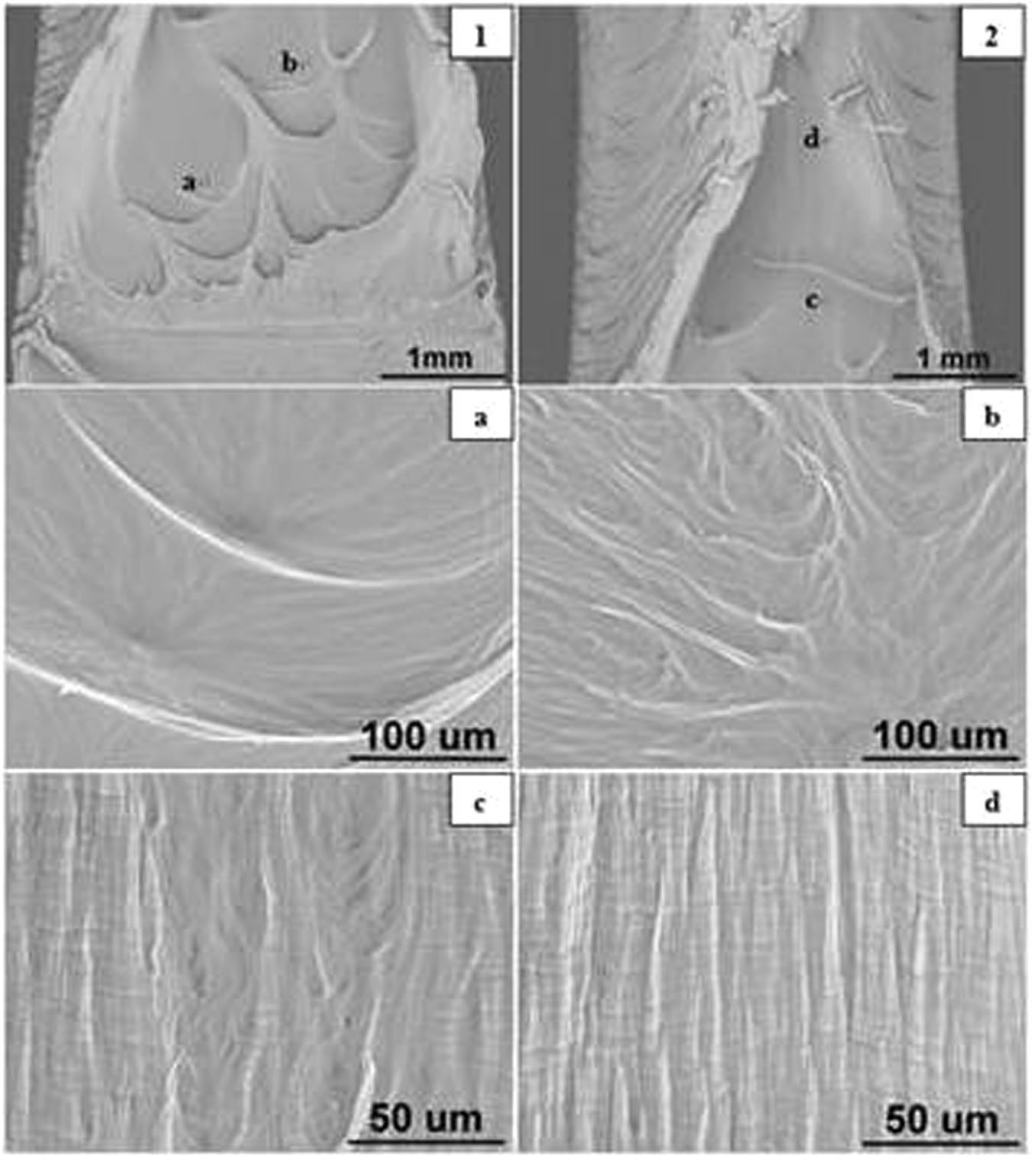
SEM images of blunt notched HDPE fracture surfaces at 50.0 mm/min (1) notch at the bottom, crack propagating from bottom to top; (2) top of propagation at 25 °C.

**Figure 14:**
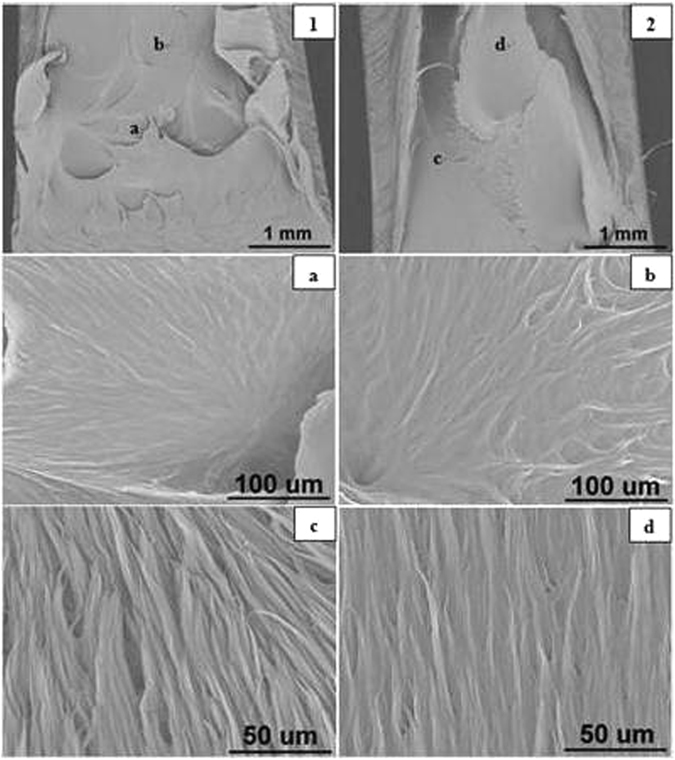
SEM images of blunt notched HDPE fracture surfaces at 100 mm/min (1) notch at the bottom, crack propagating from bottom to top; (2) top of propagation at 25 °C.

**Figure 15:**
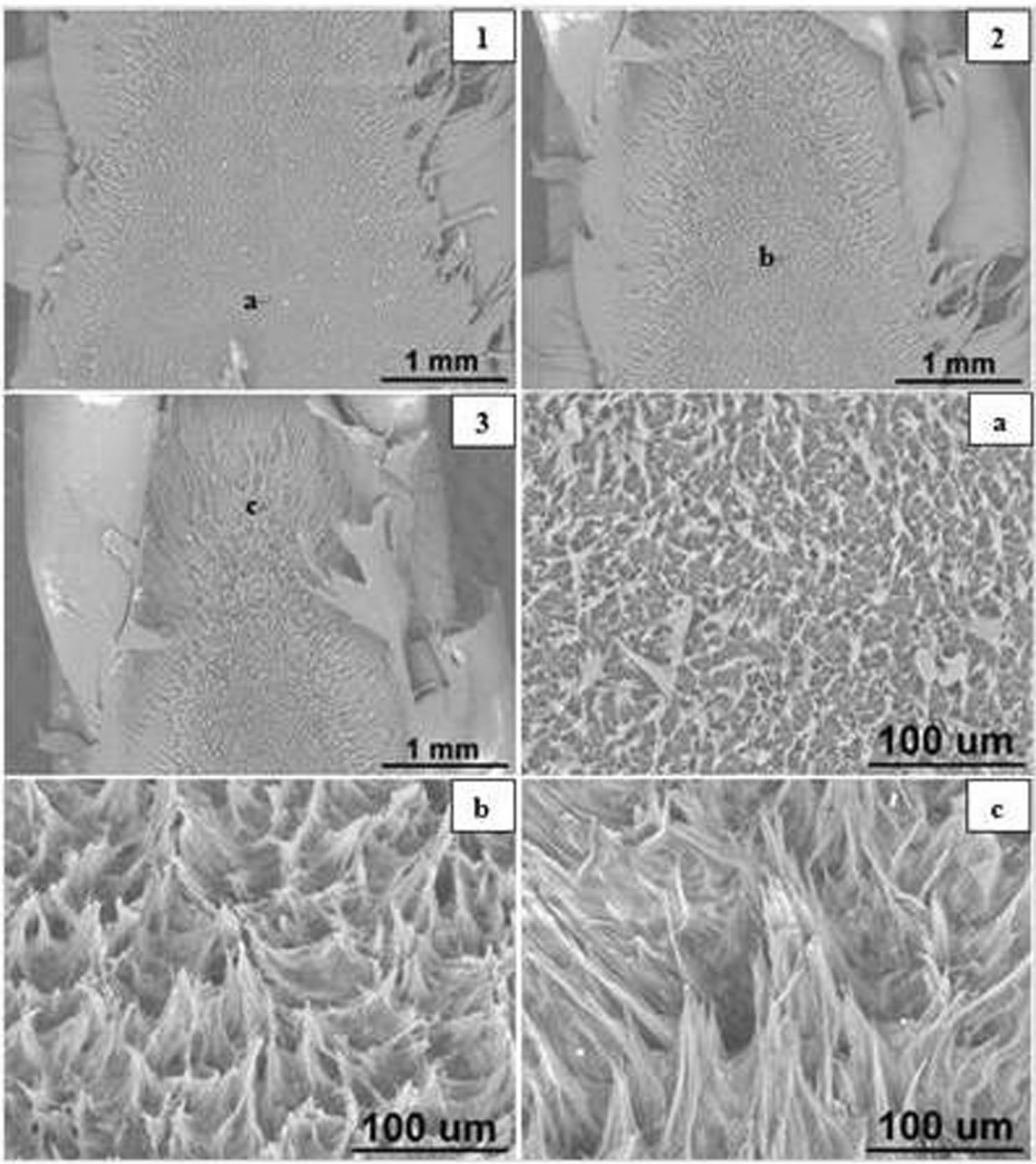
SEM images of sharp crack HDPE fracture surfaces at 0.05 mm/min (1) crack tip at the bottom, crack propagating from bottom to top; (2) medium propagation; (3) top of propagation at 25 °C.

The SEM images of blunt notched HDPE fracture surfaces at all crosshead speeds are presented in Figs 11 to 15 respectively. Photographs 1 and 2 of each figure respectively show initial (slow) and later (fast) crack propagation. Letters a, b, c, and d on photographs 1 and 2 corresponding to different crack regions are represented at a higher magnification in the photographss a, b, c and d. Overall, it has been found that specimens are ductile fractured and there are two main types of microsructures corresponding to two different fracture mechanisms over the range of crosshead speeds.

At 0.05 and 0.5 mm/min, the microsructures of fracture surfaces exhibit craze fibrils which become larger and longer as crack propagates. As can be seen in Fig. 11, craze fibrils in photograph ‘a’ are short and small with relatively average distribution, whilst in photographs ‘c’ the fibrils become longer and with larger sizes. Similar results are seen in Fig. 12 corresponding to crazing fracutre mechanism (Bandyopadhyay & Brown^12, 13^.

However, at, 50 and 100 mm/min, specimens are fractured by shear yielding mechanism with a dimple pattern. Dimple pattern corresponds to microcavities responsible for initiating crack formation. Figure 13 photographs ‘a’ and ‘b’ and Fig. 14 photographs ‘a’ and ‘b’ represent the magnified dimples. The sizes of dimples vary and the dimples are unevenly distributed on the fracture surface. But it appears that at 50 and 100 mm/min, the fracture surfaces have more larger dimples. In addition to the dimples, there are smooth fibrils which look different from crazing frcture at 0.05 and 0.5 mm/min, as can be seen in Fig. 13 photographs ‘c’ and ‘d’ and Fig. 14 photographs ‘c’ and ‘d’.

#### Sharp crack Specimens

**Figure 16:**
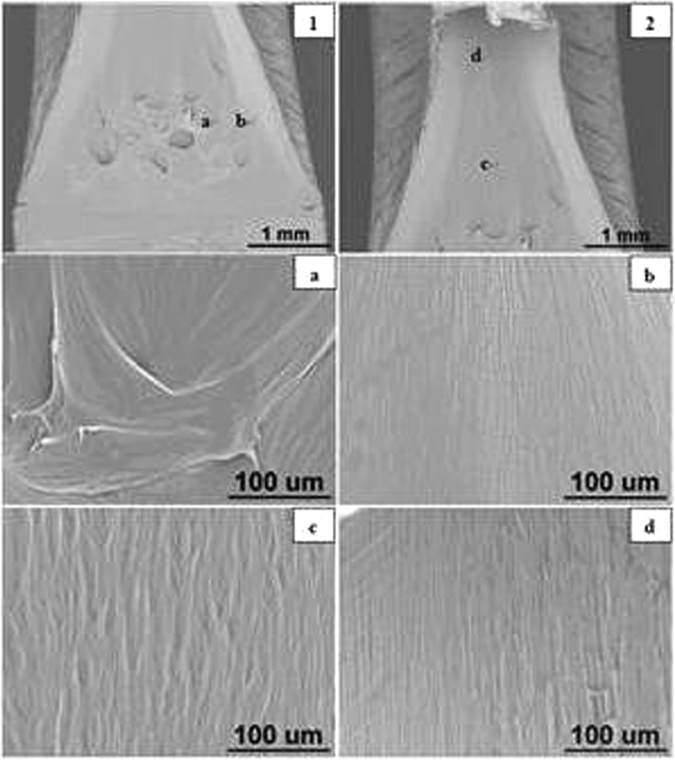
SEM images of sharp crack HDPE fracture surfaces at 5 mm/min (1) crack tip at the bottom, crack propagating from bottom to top; (2) top of propagation at 25 °C.

**Figure 17:**
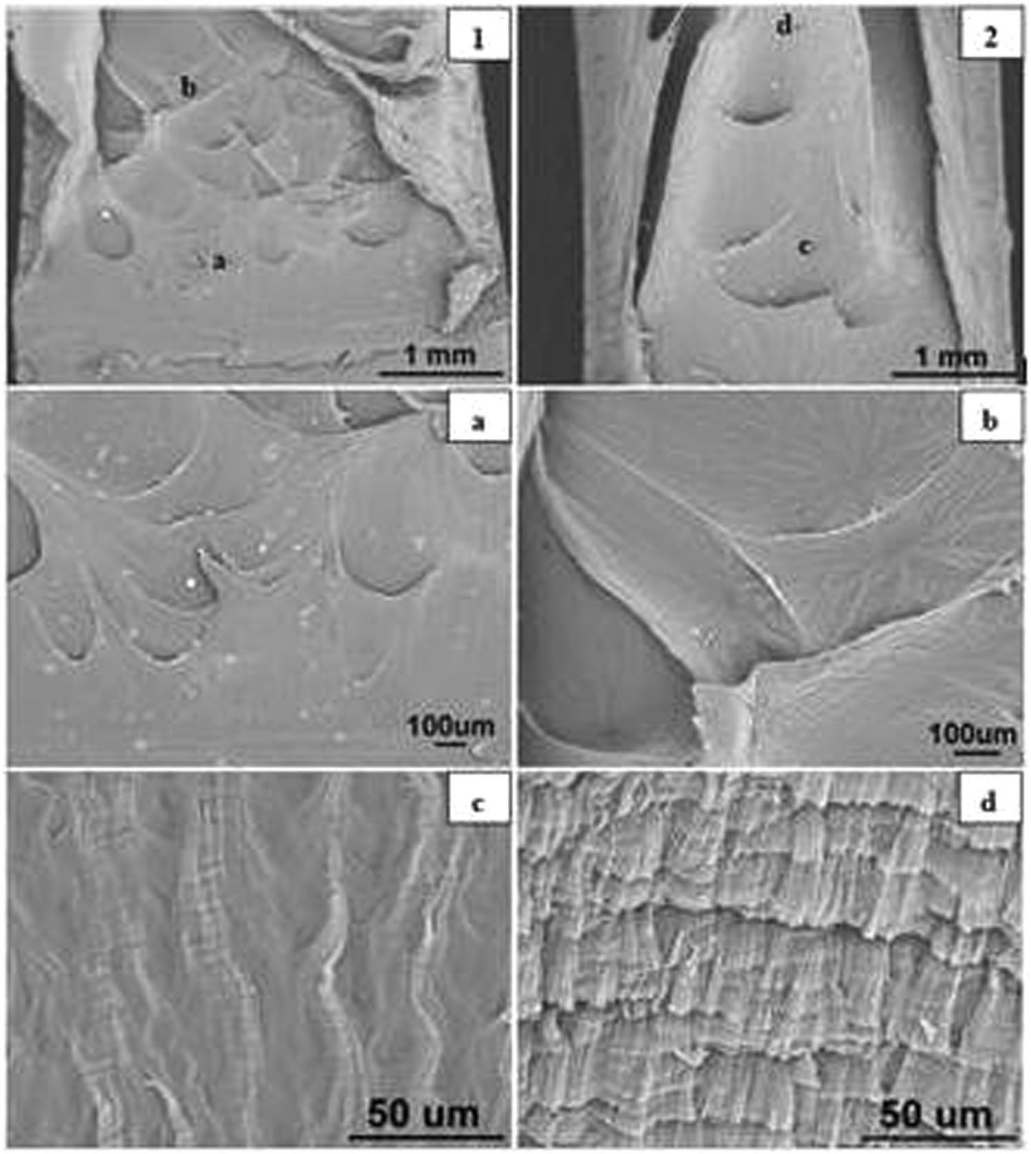
SEM images of sharp crack HDPE fracture surfaces at 100 mm/min (1) initial crack tip at the bottom, crack propagating from bottom to top; (2) top of propagation at 25 °C.

The SEM images of sharp notched HDPE fracture surfaces at crosshead speeds of 0.05, 0.5, 5, 50 and 100 mm/min are illustrated in Figs 15 to 17 respectively. Photographs 1 and 2 of each figure respectively show initial (slow) and later (faster) crack propagation. Letters a, b, c, and d on photographs 1 and 2 correspond to photographs a, b, c and d of each figure. Similar to blunt notched specimens, sharp notched specimens are also ductile fractured and there is a crazing-shear yielding fracture mechanism transition as crosshead speed increases from 0.05 to 100 mm/min.

At 0.05 and 0.5 mm/min, the microsructures of fracture surfaces are in form of craze fibrils and the fibrils become larger and longer as the crack propagates (Bandyopadhyay & Brown^12, 13^, Bandyopadhyay^20^ Bandyopadhyay & Brown^21^. As can be seen in Fig. 15, photographs ‘a’, ‘b’ and ‘c’ respectively represent initial, medium and final propagation and those fribils become longer and more significant. Similar results are seen in Fig. 16 which correspond to crazing fracutre mechanism.

By comparison, it has been observed that there is a crazing-shear yielding mechanism transition as crosshead speed increases from 0.05 to 100 mm/min regardless of blunt notch or sharp crack HDPE. Thereof crazing fracture occurs at 0.05 and 0.5 mm/min with craze fibrils, whilst shear yielding mechanism appears in fracture surfaces of specimens tested at 5, 50 and 100 mm/min with a dimple pattern as seen in Fig. 17.

## Conclusions

Influence of strain rates and temperatures on the failure and yield strength in blunt and sharp notched samples of high density polyethylene samples has been established.

1. There was no significant difference between the failure behaviour of blunt notch and sharp crack samples, indicating blunting of crack tip in sharp crack samples.
2. Both blunt notch and sharp crack HDPE are ductile fractured at crosshead speeds of 0.05, 0.5, 5, 50 and 100 mm/min. This is attributed to the failure occurring at temperatures 25 c and 45 C which are above the glass transition state of the HDPE past the brittle state of the polymer.
3. Failure and yield properties of both blunt notch and sharp crack HDPE increase as crosshead speed increases and they have a linear relation to the natural logarithm of crosshead speed.
4. Critical crack length of both blunt notch and sharp crack HDPE samples reduced exponentially as the natural logarithm of crosshead speed increases.
5. Microstructures of both blunt notch and sharp crack HDPE fracture surfaces at CHS 0.05 and 0.5 mm/min showed craze fibrils, whilst dimple pattern existed at CHS of 5, 50 and 100 mm/min.
6. Crazing to shear yielding transition occurred at CHS values between 0.5 and 5 mm/min.
7. Yield strength and the fracture strength were higher at 25 C than those at 45 C.
8. The trend for fracture strength vs ln CHS was similar for a sharp crack sample at both the temperature studied, but not for the blunt notch samples.
